# Equity effect of a community-based primary healthcare program on the incidence of childhood morbidity in rural Northern Ghana

**DOI:** 10.1017/S1463423625000106

**Published:** 2025-02-28

**Authors:** Edmund Wedam Kanmiki, Abdullah A. Mamun, James F. Phillips, Martin O’Flaherty

**Affiliations:** 1 Poche Centre for Indigenous Health, Faculty of Health, Medicine and Behavioural Sciences, The University of Queensland, Brisbane, QLD, Australia; 2 ARC Centre of Excellence for Children and Families over the Life Course (The Life Course Centre), The University of Queensland, Brisbane, QLD 4068, Australia; 3 Heilbrunn Department of Population and Family Health, Mailman School of Public Health, Columbia University, New York, NY, USA; 4 School of Human Movement and Nutrition Sciences, Faculty of Health, Medicine and Behavioural Sciences, The University of Queensland, Brisbane, QLD, Australia

**Keywords:** Child health, childhood morbidity, community-based healthcare, equity, morbidity, under-five

## Abstract

**Background:**

Childhood morbidity is a precursor and contributor to under-five child mortality. Community-based primary healthcare programs are culturally responsive and low-cost strategies for delivering maternal and child health services in rural communities.

**Aim:**

To evaluate the equity effect of the Ghana Essential Health Intervention Program (GEHIP) – a five-year community-based primary healthcare program – on childhood morbidity.

**Methods:**

GEHIP was implemented in the Upper East region of Northern Ghana. Household baseline and end line surveys conducted in 2010/2011 and 2014/2015, respectively, from both intervention and comparison districts were used to assess three childhood morbidity conditions: maternal recall of neonatal illness, the incidence of diarrhoea, and fever. Difference-in-differences analysis, mean comparison test, and multivariate logistic regressions are used to assess the effect of GEHIP exposure on these three childhood morbidity conditions.

**Results:**

Baseline sample data of 2,911 women and end line sample of 2,829 women were included in this analysis. There was generally more reduction in all three childhood morbidity conditions in intervention communities relative to comparison communities. Diarrhoea and fever had a statistically significant treatment effect (AOR = 0.95, p-value<0.01 and AOR = 0.94, p-value<0.001). Results of equity analysis indicate significant mean reductions for both the poor and non-poor for neonatal illness and diarrhea, while only the intervention group had a significant reduction for both poor and non-poor for fever. Regression analysis shows no significant equity/inequity effects of GEHIP on the incidence of diarrhoea and fever. Neonatal illness, however, shows significant effects of wealth within the intervention group.

**Conclusion:**

This study shows that GEHIP contributed significantly to childhood morbidity reduction. This implies that community-based strategies have the potential to improve child health and contribute to the attainment of the United Nations sustainable development goal related to child health. Specific targeted measures are recommended to ensure both the poor and relatively better-off benefit from interventions.

## Background

Neonatal illness, diarrhoea, and fever are among the major causes of under-five child mortality (Liu *et al.*, 2016; World Health Organization, 2017). Global under-five mortality rates have seen a significant decline of approximately 61% between 1990 and 2020 (United Nations, 2023). Despite this progress, it is estimated that in the year 2020 alone, about 13,800 under-five deaths occurred each day on average (United Nations, 2023). It is further projected that between 2017 and 2030, about 10 million children will lose their lives from preventable causes before reaching their fifth birthday (UNICEF, 2018). A high portion of child mortality is attributable to the neonatal period, where approximately half of all under-five deaths (2.4 million) occur (United Nations, 2023).

Sub-Saharan Africa and South Asia account for the highest burden of child mortality. For instance, about 83% of all under-five deaths in 2022 occurred within sub-Saharan Africa (58%) and South Asia regions (25%) (United Nations, 2023). The current United Nations Sustainable Development Goals (SDGs) specifically target reductions in under-five mortality to 25 per 1000 live births and 12 per 1000 live births for neonatal mortality. However, projections suggest that the SDGs targets are likely not to be achieved by 2030 unless there is a rapid and strategic investment in child survival, particularly in the sub-Saharan Africa region and conflict-affected areas (d’Harcourt et al 2017; Moyer and Hedden, 2020). This underscores the need for proven context-specific interventions that address the underlying causes of poor child health. Thus, critical analyses of programs and strategies are needed to inform countries striving to achieve the SDG mortality reduction targets (Hansen and Schellenberg, 2016; Moyer and Hedden, 2020).

Evidence shows there exist inequalities in childhood mortality and morbidity. For instance, in the year 2022, under-five mortality rate among children in the poorest households ranged from 3 per 1,000 live births to as high as 142 per 1,000 live births, while those in the richest ranged from 2 per 1,000 live births to 95 per 1,000 live births (United Nations, 2023). Indeed, studies have shown that preventable maternal and child deaths emerge from a complex interplay of economic and socio-cultural barriers that limit access to a reasonable standard of healthcare (Oduro-Mensah *et al.*, 2013). Even within countries, inequalities exist across socio-economic strata that are linked to inequities in access to healthcare services (Kanmiki *et al.*, 2014; Boerma *et al.*, 2018; United Nations, 2023).

Ghana is not an exception to this problem. In Ghana, under-five mortality is 42 per 1000 live births. It is higher among the poorest households (53 deaths per 1000 live births) compared to the richest households (32 deaths per 1000 live births) (United Nations, 2023). Childhood mortality is also disproportionally distributed among the regions of Ghana with the Greater Accra region having the lowest rate of 33 deaths per 1000 live births, while the northern region (one of Ghana’s poorest regions) has the highest under-five mortality rate of 61 deaths per 1000 live births (United Nations, 2023).

Research from several countries shows that children from poor and low socio-economic backgrounds have a higher prevalence and clustering of childhood morbidity conditions including fever, diarrhoea, acute respiratory tract infections (ARI), and pneumonia (Winskill *et al.*, 2021; Rahman and Hossain, 2022; Wang *et al.*, 2022). A study of 39 LMICs revealed high inequities in these morbidity conditions among children from poor households, those with limited access to healthcare services, and those living in rural and remote communities have elevated risks (Winskill *et al.*, 2021). In Bangladesh, children from low socio-economic status households, low parental education, and those without access to safe drinking water and hygienic toilet facilities and rural residents had the highest risk of suffering diarrhoea, fever, and ARI (Rahman and Hossain, 2022).

Community-based primary healthcare programs are known to improve access to health services for mothers and children in rural poor and hard-to-reach communities leading to some improvements in health outcomes (Macinko, Starfield and Erinosho, 2009; Gilmore and McAuliffe, 2013; Lu *et al.*, 2020). They have been associated with higher rates of immunization coverage, exclusive breastfeeding, use of oral rehydration therapy, contraceptive knowledge and use, understanding of basic hygiene, and management of diarrhoea and other common diseases in children (Emond *et al.*, 2002; Macinko *et al.*, 2007). Therefore, community-based primary healthcare programs could also have a positive effect on childhood morbidity and improve equity in the incidence and prevalence of morbidity conditions.

Since child health outcomes can vary significantly across different sub populations within a country, evaluating the equity effects of community-based primary healthcare programs on child health outcomes is important to guide policymakers in low- and middle-income countries (LMICs) in making informed decisions about resource allocation and program design (Kanmiki et al., 2023). This ensures that these programs effectively reach all children in need and contribute to a more equitable distribution of health outcomes across communities. Infant mortality reductions associated with the delivery of care through community-based health programs are said to be about 40% on average across studies, with some interventions reporting as high as 71% reductions in infant mortality (Macinko, Starfield and Erinosho, 2009). Particularly, community-based programs are effective in preventive interventions for maternal and child healthcare (Gilmore and McAuliffe, 2013). A study in Brazil found that a community-based program reduced infant mortality by almost half, and this reduction was larger in rural and poorer regions compared to developed regions which already had relatively better rates (Macinko *et al.*, 2007).

Community-based primary healthcare programs hold promise for improving child health outcomes in deprived settings. Studies have shown their effectiveness in reducing morbidity and mortality compared to mainstream care (Lewin *et al.*, 2010). However, a crucial question remains: Do these programs contribute to a more equitable distribution of health across communities? Evaluating the impact on health equity is essential, particularly in the context of achieving the sustainable development goals (SDGs), which emphasize leaving no one behind (United Nations, 2016). This study aims to contribute to this evidence by assessing the equity effects of a community-based healthcare program – the Ghana Essential Health Interventions Program (GEHIP) – on three common under-five health conditions: neonatal illness, diarrhoea, and fever. We focused on these three prevalent childhood illnesses (neonatal illness, diarrhoea, and fever) for this study because they are among the leading contributors to under-five mortality (Liu *et al.*, 2016; World Health Organization, 2017).

## Description of GEHIP’s intervention

GEHIP was a five-year health system strengthening program implemented to demonstrate practical means of scaling-up Ghana’s Community-based Health Planning and Services program (CHPS) and introducing improvements to the range of services provided by community health workers (Awoonor-Williams *et al.*, 2013). CHPS was made a national program in the year 2000 following successful field trials that demonstrated its ability to improve access to primary healthcare for remote communities and lead to improvements in a range of maternal and child health indicators (Awoonor Williams, Phillips and Bawah, 2019; Kanmiki *et al.*, 2019). However, a decade after it had been made a national policy, scale-up was slow and limited by a range of technical, logistical and financial constraints for scaling-up coverage at the district levels. The GEHIP program was therefore implemented in response to these healthcare delivery challenges in Ghana.

Seven districts in the Upper East region of northern Ghana were involved in the GEHIP project, three served as intervention districts and four other contiguous districts served as comparison districts. Both intervention and comparison districts were chosen because of their similar socio-economic characteristics. At the time of implementation, these districts were ranked among the poorest 5% in Ghana with an average per capita income of roughly a quarter of the national average (Kanmiki *et al.*, 2019). GEHIP’s interventions included training and technical assistance provided to district-level health managers and frontline community health workers. These trainings aimed at building their capacity in both community and stakeholder engagement to support health service delivery and utilization of maternal and child health services including antenatal care, skilled delivery, personal hygiene, exclusive breastfeeding, and good nutrition during pregnancy as well as post-partum, etc. (Kanmiki *et al.*, 2023). As a health system strengthening initiative, no new modalities were employed. Instead, the project focused on the challenge of effectively marshalling the system associated with the management of existing staff, equipment, pharmaceutical supplies, and leadership capacity for primary healthcare. Focus was directed on improving the implementation of each of the WHO’s six pillars of health system functioning (World Health Organization, 2007). At the onset of the GEHIP, there was no shortage of nurses for expanding community-based healthcare operations in Ghana; but rather, a lack of health facilities in most communities where trained nurses could be posted to render services (Kanmiki et al., 2019). Also limited was district-level leadership’s understanding of strategies for obtaining resources for constructing and managing community health posts effectively (Awoonor-Williams *et al.*, 2013).

To address these challenges, GEHIP developed a strategic framework for strengthening community-based primary healthcare. The strategy was focused on improving district-level leadership capacity, use of information for decision-making, logistics, budgeting, health worker training, and deployment for the provision of healthcare at community locations (Kanmiki et al., 2023). Specific maternal and child health interventions were included within GEHIP, including the integrated management of childhood illness regimen recommended by the WHO (World Health Organization, 2013).

GEHIP also developed a referral service program that enhanced health facility delivery using community engagement strategies to improve social support for referral operations (World Health Organization, 2013). In the programmatic context of the Ghana Health Service (GHS), region-wide implementation of some interventions involving health worker training and deployment program focused on WHO recommendations for caring for the mother and newborn as well as the integrated management of childhood illness (World Health Organization, 2005, 2013). All such national program interventions were implemented equivalently in treatment and comparison districts. More details of GEHIP’s interventions are provided in Supplementary Table 1.

## Methods and material

### Analytical framework

**Figure 1.**
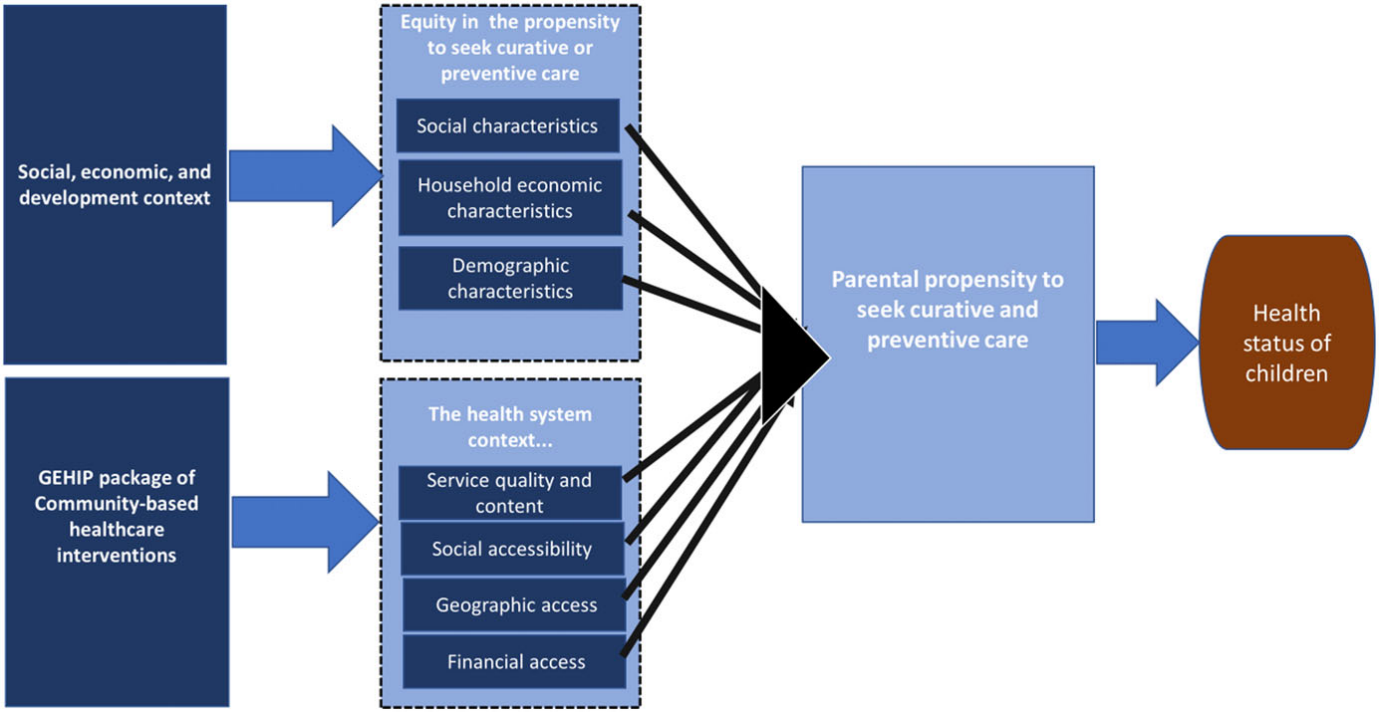
Analytical framework of the study.

Figure 1 presents the analytical framework guiding the implementation of this study. Based on the social determinants of health theory, structural determinants such as socioeconomic, cultural, and political context influence household economic status, social norms, and other circumstances, which in turn interact with intermediate determinants of health including behavioural factors, health system characteristics, and context and access to healthcare services (World Health Organisation, 2010). GEHIP package of community-based healthcare programmes was modelled to improve both the demand and supply side factors of health seeking and delivery in remote communities through improvements in service quality, social accessibility, geographical access, and impact on financial access as well (Kanmiki *et al.*, 2019). This study posits that these approaches to health service enhancement could alter the potential negative effects of some of the social determinants of health and improve parental propensity to seek healthcare service and by so doing improve the health of under-five children.

### Study design and data collection

GEHIP was a plausibility trial in that the introduction of its interventions was configured at the district level which prevented the imposition of randomized sampling of observational units (Habicht, et al 1999; Victora et al., 2004). Methods for statistical analysis of non-experimental conditions were therefore required (Heckman and Hotz, 1989). Therefore, a quasi-experimental study design approach was used involving two rounds of household women surveys at baseline (in 2010/2011) and end line (in 2014/2015). A two-stage sampling approach was applied. In the first stage, 66 predominantly rural enumeration areas (EAs) were first drawn from the study districts. This was then followed by the second stage of sampling, which involved a random sampling of households proportional to the population size of each study district (Bawah *et al.*, 2019). In each sampled household, all resident women of reproductive age (15–49) were eligible to be interviewed.

**Figure 2.**
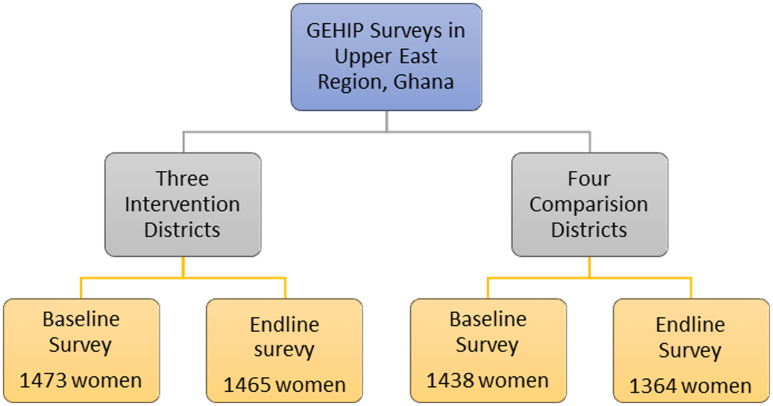
Study participants from GEHIP surveys used in this study.

The household demographic and health survey (DHS) questionnaire was adopted and used for both baseline and end line surveys (DHS, 2022). Prior to data collection, the adopted questionnaire underwent pretesting with a small sample to ensure its clarity and relevance. The surveys collected data on women’s demographic and socio-economic characteristics, including their birth histories, access to care, healthcare utilization, and contraceptive use, among others. Both study rounds were done using the same enumeation areas (EAs) although no efforts were made to interview the same women at end line. 5604 women from 4378 households were interviewed at baseline, while 5914 women from 4421 households were interviewed at end line. This study uses data from a subset of this sample for women who have had birth within the last five years and provided answers on their child morbidity. After accounting for this restriction, the baseline included 2,911 women, of whom 1,473 were from intervention areas and 1,438 were from comparison districts. The end line sub-sample includes 2,829 women, of whom 1,465 were from intervention and 1,364 from comparison districts. No missing data challenges were encountered. Figure 2 presents study participants by intervention and comparison districts.

### Measures

Three childhood morbidity outcome variables have been examined in this study. Neonatal illness, diarrhoea within the last two weeks, and fever within the last two weeks prior to the interview. All three variables were part of the information collected during the household surveys from women on their most recent birth below five years of age. A child had neonatal illness if the mother or primary caregiver responded yes to a question seeking to know if the child was sick within the first 28 days/month after delivery. A child was considered to have diarrhoea if the child had three or more watery stools or blood in stools within the last two weeks before the survey. A child was considered to have a fever if the mother or primary caregiver reported that within the last two weeks before the survey, the child had a high temperature or shivering for a period that led to seeking healthcare or administering treatment.

Potential confounding variables that have been controlled for in multivariate analysis are mother’s age group (categorized into 15–19, 20–34, and 35–49), marital status (single, married), mother’s highest educational attainment (no formal education, primary/JHS/Middle school), religious affiliation (Christianity, Traditional African religion, and Islamic religion), location of residence (urban, semi-urban, rural), parity (categorized into ‘one birth’, ‘2-4 births’, and ‘5 to 7 births and 8 or more births’). We did not find respondents who did not belong to any of the three main religions in Ghana. Our categorization of location of residence into three (urban, semi-urban, and rural) is in recognition of the fact that settlements in developing countries like Ghana are not strictly dichotomous (Mcgranahan and Satterthwaite, 2014). The semi-urban category therefore represents settlements that has a semblance of urbanization but could not qualify as being urban due to limited social amenities, population, and physical infrastructure.

Socio-economic status was measured using the household wealth index and the mother’s educational attainment as equity measures. In this study, as equity stratifiers, we categorize the wealth index variable into two categories (poor and non-poor) and the educational attainment variable into ‘some formal educated’ and ‘no-formal education’.

### Statistical analysis

Descriptive statistics are first used to show the proportion of under-five childhood morbidity before and after GEHIP’s community-based intervention in the two arms of the study and the proportion distributed by household wealth index and mother’s education. To estimate the impact of GEHIP’s community-based health program on childhood morbidity, the Heckman difference-in-differences (DID) is applied to estimate its average treatment effects (Heckman, 1974; Heckman and Hotz, 1989).

Furthermore, t-tests are used to assess the significance of mean differences between the ‘poor’ and the ‘non-poor’ as well as between those with some formal education and those without formal education on all three outcome variables. Then binary logistic regression models with two and three-way interaction terms are used to examine the equity effect of GEHIP’s community-based health program on outcome variables. To ease interpretation, we present post-estimation marginal effects expressed in percentage points. Full regression tables are included in the appendix. Equation (1) shows the specification of the logistic model for estimating the effect of wealth status:
(1)

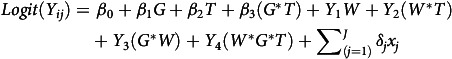




where ***Y*** is the binary outcome indicator for individual i at time t (taking the values 1 where child morbidity is present and 0 otherwise). **G** is an area indicator for treatment districts (G = 1) and zero otherwise. **T** is a dummy variable defining survey time, T = 1 for the end line and 0 for baseline observations. **β** is the intercept and regression coefficients. **W** is the household wealth index. The **γ** parameters represent adjusted effects of wealth in comparison districts at baseline (**γ**_**1**_), the change in the effect of wealth in comparison districts between baseline and end line (**γ**_**2**_), the difference in the effect of wealth between intervention and comparison districts (**γ**_**3**_) at baseline, respectively. Thus, **γ**_**4**_ estimates the effect of GEHIP on health equity relative to comparison districts, which is the difference in the change in equity between intervention and comparison districts. The vector **X** refers to **J** control variables in the model. For each outcome indicator of interest, separate models are fitted for wealth and educational attainment. For models using educational attainment as the equity stratifier, the wealth index is replaced with education in equation (1). STATA software was used in all the analyses.

## Ethical considerations

Ethics approval for this study was obtained from the Human Ethics and Research Office of the University of Queensland No: 2020000457. The study uses secondary data from GEHIP project which was granted ethical approval by the Navrongo Health Research Centre Ethics Review Board in Ghana under IRB number FWA00000250. Written informed consent was obtained from all participants prior to interviews by the GEHIP research team. Only de-identified data have been used in the analysis for this paper.

## Results

Table 1 presents the background characteristics of the study sample. At baseline, around 3% were teenagers, while 51% and 46% were 20–34 years and 35–49 years old, respectively. At end line, however, the majority of the study sample was between 20 and 34 years old (68%) with not much difference between intervention and comparison districts. As high as 88% of respondents were married at baseline and 91% of respondents were married at end line, again not much difference was observed for the two arms of the study. As a whole, respondents without formal education reduced from 76% at baseline to 63% at end line. Also, respondents associated with the Christian religion increased from 51% at the baseline to 57% at end line. As high as 87% of the study sample were residents in rural areas at baseline while 78% were residents in rural areas at end line. Chi-square test shows the proportion of most characteristics were statistically different between intervention and comparison districts for both surveys.

**Table 1.** Background characteristics: chi-square comparison at baseline and end line (intervention vs. comparison)

		Baseline Survey						End line Survey				
		Intervention		Comparison				Intervention		Comparison		
Descriptive Statistics		N	%	n	%	X^2^ P-value		P-value	%	n	%	X² P-value
**Age group**	15–19	67	3.3	69	3.4	0.286		87	5.9	98	7.2	0.001
	20–34	1,012	49.9	1,059	52.3			963	65.7	964	70.7	
	35–49	948	46.8	897	44.3			415	28.3	302	22.1	
**Marital status**	Single	285	13.9	226	10.9	0.004		134	9.2	119	8.7	0.694
	Married	1,773	86.2	1,847	89.1			1,331	90.9	1245	91.3	
**Education**	No formal education	1,498	72.7	1,658	79.9	<0.001		884	60.3	912	66.9	<0.001
	Prim/JHS/middle sch	487	23.6	371	17.9			444	30.3	358	26.3	
	Sec/tertiary	75	3.6	46	2.2			137	9.4	94	6.9	
**Religion**	Christianity	1,162	56.4	966	46.6	<0.001		865	59.0	752	55.1	0.103
	Traditional	436	21.2	446	21.5			169	11.5	178	13.1	
	Islam	461	22.4	663	32.0			431	29.4	434	31.8	
**Location of residence**	Urban	40	1.9	48	2.3	<0.001		195	13.3	37	2.7	<0.001
	Semi-urban	350	17.0	77	3.7			192	13.1	229	16.8	
	Rural	1,671	81.1	1,949	94.0			1,078	73.6	1,098	80.5	
**Wealth index (5)**	Poorest	528	25.6	352	17.0	<0.001		295	20.1	207	15.2	<0.001
	Poorer	446	21.6	434	21.0			288	19.7	244	17.9	
	Better	349	16.9	458	22.1			353	24.1	259	19.0	
	Less Poor	393	19.1	418	20.2			279	19.0	280	20.5	
	Least Poor	345	16.7	408	19.7			250	17.1	374	27.4	
**Parity**	One birth	281	13.7	306	14.8	0.064		376	25.7	370	27.1	0.313
	2–4 births	901	43.8	932	44.9			676	46.1	604	44.3	
	5–7 births	702	34.1	704	33.9			377	25.7	343	25.2	
	8 or more births	175	8.5	133	6.4			36	2.5	47	3.5	

Table 2 presents frequencies and percentages of outcome variables for baseline and end line. The proportion of under-fives who fell sick within the first month after birth (neonatal illness) reduced from 32% at baseline to 17% at end line in the intervention group. The non-intervention group also observed some reduction. The incidence of diarrhoea reduced from 17% to 10% in the intervention group, while the non-intervention group was effectively unchanged. Incidence of fever reduced from 10% to 6% in the intervention group, while the non-intervention group rather experienced a slight increase from 9% to 12% between baseline and end line, respectively.

**Table 2.** Under-five childhood morbidity for neonatal illness, diarrhea and fever

		Baseline				End line			
		Intervention		Non-Intervention		Intervention		Non-Intervention	
Indicators		N	%	N	%	N	%	N	%
**Neonatal illness**	yes	302	32	242	28	245	17	229	17
	No	642	68	620	72	1219	83	1135	83
**Diarrhoea**	yes	204	17	166	14	149	10	178	13
	No	972	83	1018	86	1284	90	1172	87
**Fever**	yes	117	10	110	9	84	6	160	12
	No	1088	90	1117	91	1337	94	1175	88

Table 3 presents the regression results of the difference in differences estimations. The interaction term represents the average treatment effects of GEHIP’s intervention. While all three indicators have a reduction in prevalence as a result of GEHIP’s intervention, diarrhoea and fever had a statistically significant effect (AOR = 0.95, p-value<0.01 and AOR = 0.94, p-value<0.001).

**Table 3.** Difference in difference treatment effects of GEHIP on neonatal illness, diarrhea and fever

	Neonatal illness		Diarrhea		Fever	
Variables	AOR	95% CI	AOR	95% CI	AOR	95% CI
Treatment *Time	0.98	(0.93 – 1.03)	0.95**	(0.91 – 0.98)	0.94***	(0.91 – 0.97)
**Treatment**	1.03	(0.99 – 1.08)	1.05**	(1.02 – 1.08)	1.02	(0.99 – 1.04)
**Time**	0.88***	(0.85 – 0.91)	0.98	(0.96 – 1.01)	1.03*	(1.00 – 1.05)
**Age Group (Compared with 15-19yrs)**						
20–34	1.02	(0.96 – 1.08)	1.01	(0.96 – 1.06)	1.01	(0.98 – 1.05)
35–49	0.99	(0.92 – 1.06)	0.99	(0.94 – 1.05)	1.01	(0.97 – 1.05)
**Marital Status (Compared with Single)**						
Married	1.01	(0.97 – 1.06)	0.97	(0.94 – 1.01)	1.00	(0.97 – 1.03)
**Educational Status (Compared with No formal education)**						
Prim/JHS/Middle Sch	1.03*	(1.00 – 1.06)	0.99	(0.97 – 1.02)	1.01	(0.99 – 1.03)
Sec/Tertiary	1.02	(0.97 – 1.08)	0.95*	(0.91 – 1.00)	1.00	(0.96 – 1.04)
**Wealth Index (Compared with Poorest)**						
Poor	0.99	(0.95 – 1.02)	1.01	(0.98 – 1.04)	1.00	(0.97 – 1.02)
Better	1.00	(0.97 – 1.04)	0.99	(0.97 – 1.02)	0.97**	(0.94 – 0.99)
Less poor	1.00	(0.97 – 1.04)	1.01	(0.98 – 1.04)	0.99	(0.97 – 1.02)
Least poor	1.00	(0.96 – 1.04)	1.03	(0.99 – 1.06)	0.99	(0.96 – 1.02)
**Religion (Compared with Christianity)**						
African Traditional Religion	0.98	(0.94 – 1.01)	0.99	(0.96 – 1.02)	0.99	(0.97 – 1.01)
Islam	1.00	(0.97 – 1.03)	1.02	(0.99 – 1.05)	1.02	(1.00 – 1.05)
**Ethnicity (Compared with Bulisa)**						
Frafra	1.03	(0.99 – 1.07)	1.06**	(1.02 – 1.09)	1.04**	(1.02 – 1.06)
Kusasi	0.93**	(0.89 – 0.97)	1.02	(0.98 – 1.05)	1.04**	(1.01 – 1.07)
Other	0.95*	(0.90 – 0.99)	1.02	(0.98 – 1.06)	1.05**	(1.02 – 1.08)
**Location of Residence (Compared with Urban)**						
Semi-urban	1.05	(0.98 – 1.11)	0.99	(0.95 – 1.04)	1.04*	(1.00 – 1.07)
Rural	1.02	(0.97 – 1.08)	0.99	(0.95 – 1.03)	1.02	(1.00 – 1.05)
**Parity (Compared with one birth)**						
2-4 births	1.00	(0.96 – 1.03)	0.96*	(0.94 – 0.99)	1.00	(0.98 – 1.02)
5-7 births	0.99	(0.94 – 1.03)	0.95*	(0.92 – 0.99)	1.01	(0.98 – 1.04)
8 or more births	1.00	(0.93 – 1.08)	0.98	(0.92 – 1.04)	0.99	(0.94 – 1.04)

Constant	1.31***	(1.19 – 1.44)	1.17***	(1.08 – 1.26)	1.02	(0.96 – 1.08)

Observations	4,604		5,097		5,142	
R-squared	0.04		0.01		0.01	

*** p < 0.001, ** p < 0.01, * p < 0.05.

Covariate that were significantly associated with treatment effects of neonatal illness are the mother’s educational status and ethnicity. Children born to families belonging to the Kusasi and other ethnic groups were significantly less likely to have neonatal illness compared with those of the Builsa ethnicity. For the prevalence of diarrhoea, the mother’s educational status, ethnicity, and parity (number of previous births) were significantly associated with the treatment effects of GEHIP on diarrhoea prevalence. Children whose mothers had up to secondary educational attainment and above had 0.95 fewer odds of having diarrhoea compared to those with no formal education (AOR = 0.95, p-value<0.05). Those belonging to the Frafra ethnicity have higher odds of diarrhoea compared with the Builsa ethnic group (AOR = 1.06, p-value<0.01). Also, children of multiparous mothers were less likely to have diarrhoea compared with those of primiparous mothers (AOR = 0.95, p-value<0.05).

For fever prevalence, wealth index, ethnicity, and place of residence were significantly associated with the prevalence of fever. Children belonging to the middle wealth quintile (better) are less likely to have fever compared with those of the poorest quintile. Also, those belonging to Frafra, Kusasi, and other ethnicity all have higher odds of having fever compared with those belonging to the Builsa ethnic group. Residents in semi-urban locations also had high odds of fever compared with urban residents (AOR = 1.04, p-value<0.05)

**Figure 3.**
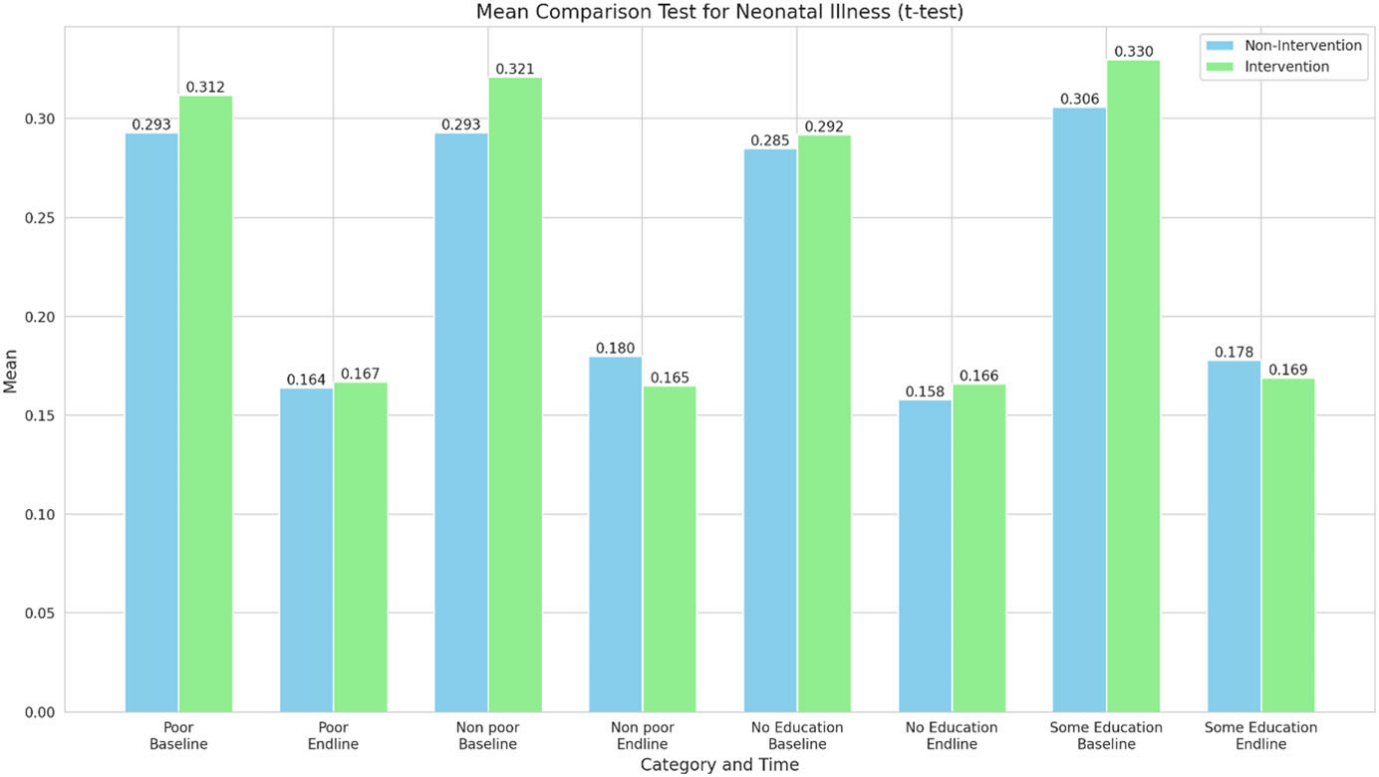
Mean comparison test of neonatal illness (t-test).

Figure 3 shows the mean comparison test results for neonatal illness between intervention and comparison groups across different categories (poor, non-poor, no education, and some education) and time points (baseline and end line). The results show a decrease in the incidence of neonatal illness from baseline to end line across all categories for both non-Intervention and intervention groups. The reduction in mean values from baseline to end line appears to be more substantial for the intervention group in most categories, suggesting a potentially positive impact of the GEHIP intervention.

**Figure 4.**
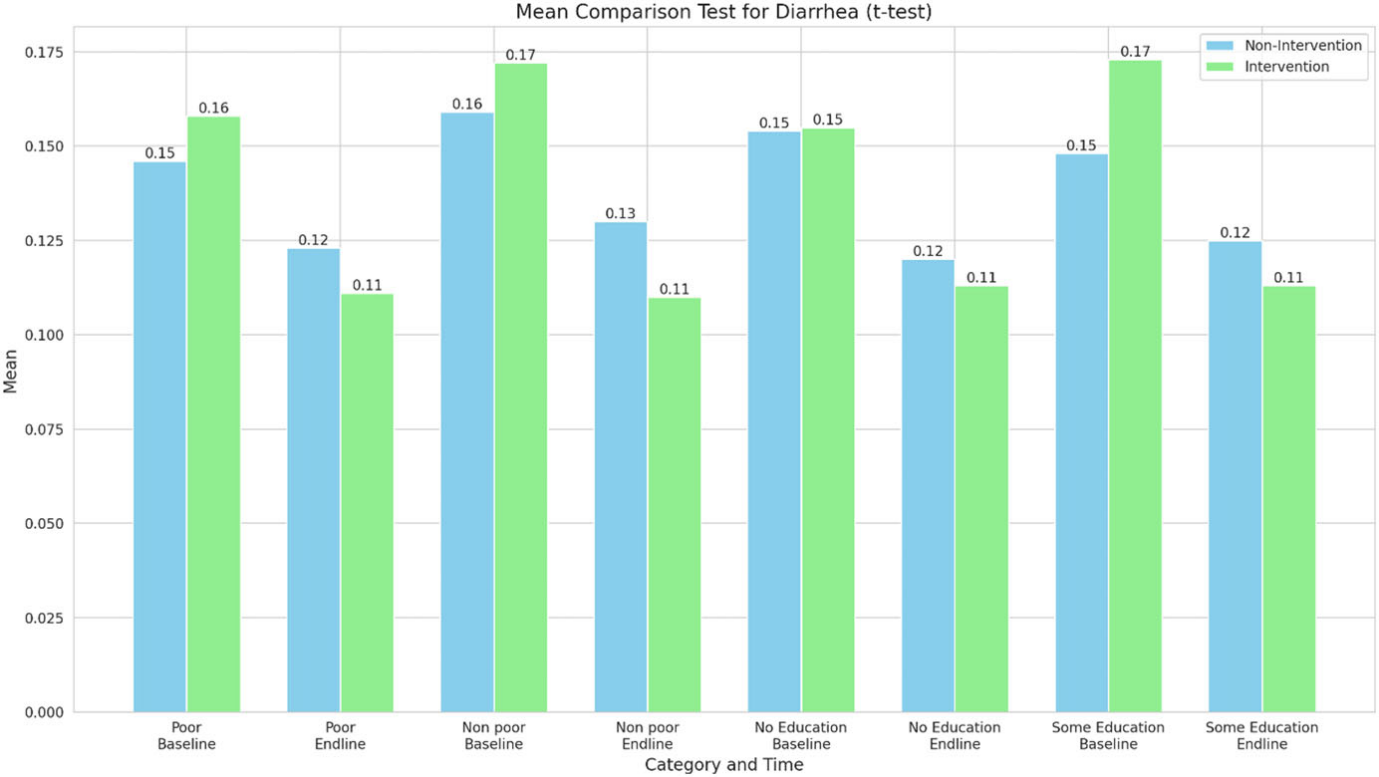
Mean comparison test for diarrhoea (t-test).

Figure 4 is the mean comparison test for the incidence of diarrhoea between intervention and non-Intervention groups across different categories (poor, non-poor, no education, and some education) and time points (baseline and end line). Small reductions over time were observed in all groups, although the intervention group seems to have a more pronounced decrease in diarrhoea incidence compared to the non-intervention group, especially for the “non poor” and “some education” categories.

**Figure 5.**
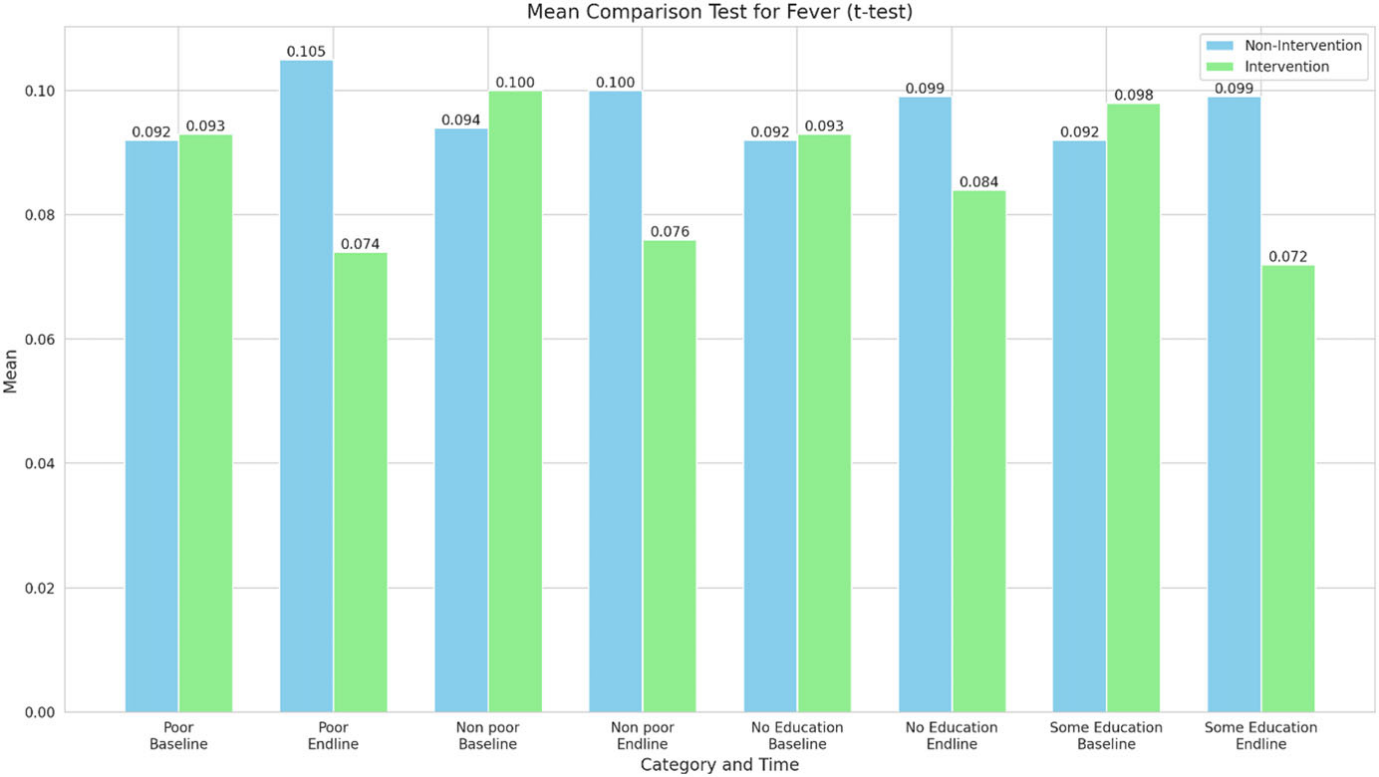
Mean comparison test for the incidence of fever.

Figure 5 shows the results of the mean comparison test for the incidence of fever. The Intervention group shows a reduction, with lower fever incidence at end line compared to baseline across all categories. The most substantial decrease is observed in the “some education” category for the Intervention group (from 0.098 to 0.072). Overall, the intervention appears to have a positive effect on reducing fever incidence across all socioeconomic and educational categories.

Table 4 presents the result of the average marginal effects of wealth index on childhood morbidity from multivariate logistic regression. Results show no significant effect of wealth index on childhood morbidity within the non-intervention groups, either at baseline or end line. For the intervention districts, no association between wealth and diarrhoea or fever was found. However, a significant positive effect of wealth (p-value = 0.012, AME = 0.062), implying that GEHIP intervention rather contributed to wealth inequality which was not existent at baseline (more details in the full regression in Supplementary Table 2).

**Table 4.** Average marginal effect of household wealth on childhood morbidity

	Delta-method					
	dy/dx	Std. Err.	z	P>z	[95% Conf.	Interval]
**Average Marginal Effects of Wealth on Neonatal illness**						
Baseline non-intervention group	0.030	0.039	0.77	0.444	−0.047	0.107
Baseline intervention group	0.010	0.040	0.25	0.804	−0.069	0.089
End line non-intervention group	−0.016	0.026	−0.63	0.527	-0.067	0.034
End line intervention group	0.062	0.025	2.53	0.012	0.014	0.110
**Average Marginal Effects of Wealth on Diarrhea**						
Baseline non-intervention group	0.053	0.030	1.79	0.073	−0.005	0.111
Baseline intervention group	0.044	0.035	1.24	0.215	−0.025	0.113
End line non-intervention group	−0.015	0.034	−0.43	0.665	−0.081	0.051
End line intervention group	0.021	0.023	0.89	0.372	−0.025	0.067
**Average Marginal Effects of Wealth on Fever**						
Baseline non-intervention group	0.027	0.020	1.330	0.185	−0.013	0.067
Baseline intervention group	0.002	0.022	0.090	0.932	−0.041	0.045
End line non-intervention group	−0.012	0.032	−0.370	0.711	−0.074	0.050
End line intervention group	-0.030	0.019	−1.530	0.127	-0.068	0.008

Table 5 also presents the average marginal effects of maternal educational attainment on our three childhood morbidity conditions. Results indicate that only the non-intervention group at baseline had a significant marginal effect of education on neonatal illness. But this was no longer the case at end line of the group. There was no marginal effect of education on diarrhoea and fever in all arms of the study.

**Table 5.** Average marginal effect of education on childhood morbidity

	Delta-method					
	dy/dx	Std. Err.	z	P>z	[95% Conf.	Interval]
**Average Marginal Effects of Education on Illness within first month after birth**						
Baseline non-intervention group	0.081	0.039	2.09	0.037	0.005	0.158
Baseline intervention group	0.078	0.048	1.64	0.101	−0.015	0.172
End line non-intervention group	−0.034	0.030	−1.11	0.267	−0.093	0.026
End line intervention group	0.059	0.030	1.94	0.052	−0.001	0.119
**Average Marginal Effects of Education on Diarrhea**						
Baseline non-intervention group	0.036	0.026	1.36	0.175	−0.016	0.088
Baseline intervention group	−0.012	0.037	−0.32	0.748	−0.085	0.061
End line non-intervention group	−0.004	0.036	−0.12	0.901	−0.074	0.065
End line intervention group	0.001	0.030	0.02	0.981	−0.058	0.059
**Average Marginal Effects of Education on Fever**						
Baseline non-intervention group	0.040	0.023	1.75	0.081	−0.005	0.086
Baseline intervention group	0.013	0.021	0.60	0.547	−0.028	0.054
End line non-intervention group	−0.008	0.029	−0.27	0.786	−0.064	0.048
End line intervention group	−0.018	0.015	−1.18	0.237	−0.048	0.012

Results of both univariate and multivariate analysis indicate a reduction in the three morbidity conditions irrespective of either the intervention or non-intervention group. However, these outcomes were not affected by equity stratifiers in the case of wealth index or educational attainment of the child’s mother.

## Discussion

This study assesses the effect and equity effect of GEHIP’s community-based health program on childhood morbidity in rural northern Ghana. Childhood morbidity contributes to mortality and has high implications on the cost of seeking health care by households as well as the health system cost of treatment. Therefore, understanding the effect of health programs on childhood morbidity is critical for the overall evidence required for improving child health and survival.

Our results show that for all three conditions, children in GEHIP’s community-based health program intervention group had more morbidity reduction compared to children in the comparison group. DIDs estimates show significant reduction effects in the prevalence of diarrhoea and fever as a result of GEHIP’s community-based primary health program. Factors such as mother’s education, ethnicity, marital status, and parity were found to be associated with childhood morbidity.

Apanga et al using a recent nationally representative dataset in Ghana found the prevalence of diarrhoea among under-fives to be 17% (Apanga and Kumbeni, 2021). This is slightly higher than observed in this study. However, this is not surprising as their results also show that children residing in rural areas had 22% lower odds of diarrhoea compared to those in urban areas. Our study is from one of the most rural and remote regions in Ghana with relatively high operations of community-based care facilities in the intervention districts at the end line and a considerable level of operation of community-based health services is the comparison districts as well.

Factors including mothers’ education, ethnicity, wealth index, and parity were significantly associated with childhood morbidity in DID estimation. These findings corroborate a previous study from Ghana that also found education and wealth index to be positively associated with lower odds of diarrhoea among under-fives (Apanga and Kumbeni, 2021). Our results indicate that when properly implemented, community-based healthcare programs could reduce childhood morbidity irrespective of individual characteristics of the study population. The fact that two determinants (ethnicity and rural/urban location of residence) were significantly associated with the mean reductions in fever prevalence may suggest that the impact of community-based health programs on child health could be altered based on geographic context of implemented communities. As these two variables are markers of the sociocultural practices and beliefs of communities, it is imperative that community-based health programs adapt to the specific nature of communities in order to achieve optimum outcomes.

This study did not find conclusive effects of GEHIP on improving equity. Results from the multivariate analysis show that there were minimal wealth or education inequalities in the incidence of diarrhoea and fever at baseline for both intervention and non-intervention groups. And this did not change at the end line of GEHIP’s intervention. With neonatal illness, results show a widening in wealth inequity for the intervention group, while the non-intervention which has significant marginal effects of mother’s educational attainment at baseline rather experienced a more even distribution at end line of the study. These results indicate that GEHIP had minimal, if any, effects on equity in childhood morbidity. Thus contradicting our initial hypothesis that GEHIP’s community-based healthcare program would have a positive effect on reducing inequalities in child health. A previous study in Malawi also found mixed outcomes on the influence of a community-based health program on newborn health indicators (Callaghan-Koru *et al.*, 2013).

One plausible explanation for the findings of this study is the fact that GEHIP was implemented in a predominantly poor region. Using household wealth index and maternal educational status as proxies for equity in such a geographically disadvantaged area may be insufficient to accurately capture varying social and economic well-being. Despite these limitations, we did observe that GEHIP effectively improved child health by reducing all three childhood morbidity conditions. This indicates that while the program may not have significantly addressed equity issues, it still had a positive impact on overall child health outcomes.

A recent study found that the average cost of treatment for an episode of fever for households in Ghana is about US$7.3, which is 4.6 times higher than the daily wage associated with unskilled labour and obviously above the average income of most rural dwellers in Ghana (Dalaba *et al.*, 2021). Thus, by contributing to reducing childhood morbidity with regards to fever, diarrhea, and neonatal illness, community-based programs also reduce the financial burden associated with health-seeking thereby contributing to the socio-economic well-being of households in rural poor communities.

The implications of these findings suggest that targeted interventions are necessary to address specific inequalities in health outcomes in disadvantaged communities even within the context of community-based healthcare programs. The study also highlights the potential limitation of using household wealth index and educational status as proxies for equity in a predominantly poor setting. Researchers may need to explore alternative measures that more accurately capture social and economic disparities in such contexts.

Despite the mixed results, this study’s findings support the community-based health planning and services policy already being implemented in Ghana, it also puts forward compelling reasons for its strengthening and adoption to fit peri-urban and poor communities in the urban settlements of Ghana and similar settings around the world. By way of policy and practice recommendations, we suggest the factoring in of tailored approaches for disadvantaged populations, strengthening community outreach and home visits by community health workers, and using simple local languages during community durbars can improve equity in health outcomes. In addition, the recently implemented community scorecard within the community-based health programme in Ghana offers a good opportunity for improving health equity (Blake *et al.*, 2016). The GHS should factor in maternal and child health equity issues within the scorecard core matrix for periodic evaluation and action.

## Study limitations and strengths

This study has limitations. First, due to pre-existing CHPS components in both intervention (25%) and comparison districts (35%) before the intervention, achieving a pure control group was not possible. However, GEHIP demonstrably accelerated CHPS uptake, with coverage reaching 85% in intervention districts compared to 55% in comparison areas. Second, inherent limitations of survey research, including recall and selection bias, measurement bias, and social desirability bias, could have influenced the results. Finally, the study’s setting is one of Ghana’s poorest regions, this might mask potential effects on equity due to the pervasive poverty in the area. Thus, there are inherent limitations with generalizing the findings of this study to other settings.

Despite these limitations, major strengths in this study include its use of data with both intervention and control groups, the use of a relatively large dataset and the application of rigorous statistical methods to partial out the group’s differences enhances the quality of evidence generated by this study. This research undoubtedly contributes to the evidence base on how community-based primary healthcare programs can improve child health equity in remote rural settings. Future research initiatives should aim to employ mixed methods to triangulate study results in order to have a comprehensive understanding of the equity effects of community-based healthcare programs. Also, efforts should be put in place to use comparison communities from geographically distinct regions to help mitigate potential contamination and ensure a more robust evaluation.

## Conclusion

This study adds to the growing evidence on the contribution of community-based health programs to child health improvement. The study shows that GEHIP’s community-based health program contributed to childhood morbidity reduction, by expanding access to primary healthcare services which mitigates the effects of household remoteness on basic preventive and curative public health care. However, we did not establish major equity effects of GEHIP in the incidence of selected morbidity conditions. This may be partly due to limitations regarding not having a pure study control and the study context.

We advocate for health policies and programs such as community-based health interventions to prioritize the needs of the poor and marginalized during design and implementation as well as routine service delivery. Ghana has since the early 2000s adopted community-based healthcare as a key policy strategy for improving primary healthcare delivery following successful phase trials in northern Ghana (Phillips *et al.*, 2020). The results of this study lend support to this policy. We urge policymakers and stakeholders to prioritize community-based healthcare programs among efforts aimed at achieving the sustainable development goals targets related to maternal and child health in Ghana and similar LMICs settings around the world.

## Supporting information

Kanmiki et al. supplementary material Kanmiki et al. supplementary material

## Supplementary material

The supplementary material for this article can be found at https://doi.org/10.1017/S1463423625000106
