# Supplementary material for: Equity effect of a community-based primary healthcare program on the incidence of childhood morbidity in rural Northern Ghana
Source: Prim Health Care Res Dev. 2025 Feb 28;26:e23. doi: 10.1017/S1463423625000106 (PMC11883786; doi:10.1017/S1463423625000106)
Supplement: Kanmiki et al. supplementary material [file S1463423625000106sup001.docx]

**Supplementary Tables**

**Table S1: The operational problems that were addressed with particular interventions by GEHIP**

| **GEHIP Interventions** | | |
| --- | --- | --- |
| **Organizational level of intervention** | **Type of operational problem to be addressed** | **Intervention** |
| **District Health Management Team** | Lack of strategic understanding of the CHPS model | Field exchanges and demonstration added to routine leadership training workshops |
|  | Lack of flexible funding | Addition of $0.85 per capita per year for three years to the District Common Fund. |
|  | Lack of evidence-based planning and budgeting | Provision of a burden of disease-based planning tool. |
| **District Hospital and Sub-district Health Centers clinical services** | - Excess neonatal mortality - Excess maternal morbidity and mortality | - Post-natal care for newborns (KMC, other interventions) - Directly Observed Therapy, Short-course (DOTS) - Emergency management: Comprehensive Emergency obstetric services to include basic Emergency obstetric care, comprehensive Emergency obstetric services, case management of newborn illnesses, comprehensive emergency referral logistics equipment, and newborn resuscitation training |
| **Sub-district Health Centers clinical services** | Lack of supervisory support for frontline providers | - Facilitative supervision training - Information for decision-making systems reform |
| **CHPS level interventions** | Lack of functioning information systems  Lack of field work, community outreach, doorstep services  Lack of organized program of referral, triage, and community engagement for emergency public health | Simplification and reform of clinical recording systems  Supervisory support for community-based care.  Comprehensive training in emergency management to include:   - Newborn surveillance and care, including resuscitation training, febrile illness recognition, and other immediate newborn monitoring - Emergency communication and referral equipment and support. - Motorcycle ambulance provision. - Volunteer ambulance driver training and deployment - Community outreach training |

**Table S2: Regression Results measuring the effect of Wealth on morbidity**

| **VARIABLES** | **Illness within first month after birth** | | **Diarrhea** | | **Fever** | |
| --- | --- | --- | --- | --- | --- | --- |
|  | **OR** | **95% Conf. Interval** | **OR** | **95% Conf. Interval** | **OR** | **95% Conf. Interval** |
|  |  |  |  |  |  |  |
| Wealth index | 1.154 | (0.797 - 1.672) | 1.580* | (0.984 - 2.536) | 1.383 | (0.847 - 2.256) |
| 1.Treatment | 1.070 | (0.655 - 1.747) | 1.662** | (1.023 - 2.699) | 1.336 | (0.741 - 2.409) |
| 0b. Treatment #co. Wealth | 1.000 | (1.000 - 1.000) | 1.000 | (1.000 - 1.000) | 1.000 | (1.000 - 1.000) |
| 1. Treatment #c. Wealth | 0.908 | (0.536 - 1.537) | 0.849 | (0.443 - 1.627) | 0.739 | (0.384 - 1.420) |
| 1.Time | 0.492*** | (0.320 - 0.757) | 1.285 | (0.761 - 2.169) | 1.798 | (0.845 - 3.823) |
| 0b. Time #co. Wealth | 1.000 | (1.000 - 1.000) | 1.000 | (1.000 - 1.000) | 1.000 | (1.000 - 1.000) |
| 1.Time#c. Wealth | 0.768 | (0.526 - 1.122) | 0.556 | (0.256 - 1.210) | 0.649 | (0.292 - 1.443) |
| 0b. Treatment #0b. Time | 1.000 | (1.000 - 1.000) | 1.000 | (1.000 - 1.000) | 1.000 | (1.000 - 1.000) |
| 0b. Treatment #1o. Time | 1.000 | (1.000 - 1.000) | 1.000 | (1.000 - 1.000) | 1.000 | (1.000 - 1.000) |
| 1o. Treatment #0b. Time | 1.000 | (1.000 - 1.000) | 1.000 | (1.000 - 1.000) | 1.000 | (1.000 - 1.000) |
| 1. Treatment #1. Time | 0.820 | (0.440 - 1.528) | 0.424*** | (0.220 - 0.814) | 0.366** | (0.142 - 0.944) |
| 0b. Treatment #0b. Time #co. Wealth | 1.000 | (1.000 - 1.000) | 1.000 | (1.000 - 1.000) | 1.000 | (1.000 - 1.000) |
| 0b. Treatment #1o. Time #co. Wealth | 1.000 | (1.000 - 1.000) | 1.000 | (1.000 - 1.000) | 1.000 | (1.000 - 1.000) |
| 1o. Treatment #0b. Time #co. Wealth | 1.000 | (1.000 - 1.000) | 1.000 | (1.000 - 1.000) | 1.000 | (1.000 - 1.000) |
| 1. Treatment #1. Time #c. Wealth | 1.890* | (0.993 - 3.597) | 1.663 | (0.639 - 4.329) | 0.820 | (0.256 - 2.625) |
| **Age group (compared with 15-19)** | | | | | | |
| 20-34 | 1.292 | (0.790 - 2.115) | 0.987 | (0.571 - 1.706) | 1.278 | (0.581 - 2.814) |
| 35-49 | 1.223 | (0.733 - 2.042) | 0.912 | (0.483 - 1.725) | 1.213 | (0.526 - 2.796) |
| **Marital Status (compared with Single)** | | | | | | |
| Married | 1.004 | (0.699 - 1.441) | 0.741* | (0.533 - 1.031) | 1.020 | (0.577 - 1.800) |
| **Education (compared with No formal education)** | | | | | | |
| Primary/JHS/Middle SCH | 1.293* | (0.993 - 1.684) | 1.078 | (0.791 - 1.469) | 1.091 | (0.853 - 1.397) |
| Secondary School+ | 1.101 | (0.725 - 1.671) | 0.830 | (0.495 - 1.394) | 0.899 | (0.486 - 1.662) |
| **Religion (compared with Christianity)** | | | | | | |
| Traditional African Religion | 0.898 | (0.634 - 1.272) | 0.919 | (0.655 - 1.288) | 0.822 | (0.552 - 1.224) |
| Islamic Religion | 0.708*** | (0.552 - 0.908) | 1.267 | (0.953 - 1.684) | 1.484** | (1.058 - 2.082) |
| **Location of Residence (compared with Urban)** | | | | | | |
| Semi-urban | 1.565 | (0.852 - 2.874) | 1.261 | (0.644 - 2.471) | 2.157* | (0.974 - 4.777) |
| Rural | 1.259 | (0.733 - 2.160) | 1.080 | (0.659 - 1.768) | 1.837* | (0.913 - 3.696) |
| **Parity (compared with one birth)** | | | | | | |
| 2 - 4 births | 1.093 | (0.817 - 1.463) | 0.763* | (0.576 - 1.010) | 1.114 | (0.746 - 1.663) |
| 5 - 7 births | 0.922 | (0.661 - 1.286) | 0.713* | (0.490 - 1.039) | 1.244 | (0.741 - 2.090) |
| 8 or more births | 0.881 | (0.485 - 1.602) | 0.765 | (0.397 - 1.474) | 1.043 | (0.429 - 2.540) |
|  | | | | | | |
| Constant | 0.264*** | (0.127 - 0.548) | 0.175*** | (0.082 - 0.373) | 0.029*** | (0.009 - 0.091) |
|  | | | | | | |
| Observations | 3,031 |  | 3,359 |  | 3,384 |  |

Robust cieform in parentheses

*** p<0.01, ** p<0.05, * p<0.1

**Table S3: Effect of Parental Education on Childhood Morbidity**

| VARIABLES | **Illness within first month after birth** | | **Diarrhea** | | **Fever** | |
| --- | --- | --- | --- | --- | --- | --- |
|  | **OR** | **95% Conf. Interval** | **OR** | **95% Conf. Interval** | **OR** | **95% Conf. Interval** |
| Education | 1.477** | (1.034 - 2.111) | 1.361 | (0.878 - 2.111) | 1.624* | (0.953 - 2.769) |
| 1.Treatment | 1.018 | (0.707 - 1.467) | 1.667** | (1.019 - 2.726) | 1.216 | (0.778 - 1.899) |
| 0b. Treatment #co. Education | 1.000 | (1.000 - 1.000) | 1.000 | (1.000 - 1.000) | 1.000 | (1.000 - 1.000) |
| 1. Treatment #c. Education | 0.974 | (0.565 - 1.680) | 0.678 | (0.341 - 1.350) | 0.709 | (0.358 - 1.403) |
| 1.Time | 0.518*** | (0.349 - 0.769) | 0.981 | (0.589 - 1.633) | 1.629* | (0.944 - 2.813) |
| 0b. Time #co. Education | 1.000 | (1.000 - 1.000) | 1.000 | (1.000 - 1.000) | 1.000 | (1.000 - 1.000) |
| 1. Time #c. Education | 0.528*** | (0.332 - 0.840) | 0.706 | (0.320 - 1.558) | 0.573 | (0.244 - 1.346) |
| 0b. Treatment #0b. Time | 1.000 | (1.000 - 1.000) | 1.000 | (1.000 - 1.000) | 1.000 | (1.000 - 1.000) |
| 0b. Treatment #1o. Time | 1.000 | (1.000 - 1.000) | 1.000 | (1.000 - 1.000) | 1.000 | (1.000 - 1.000) |
| 1o. Treatment #0b. Time | 1.000 | (1.000 - 1.000) | 1.000 | (1.000 - 1.000) | 1.000 | (1.000 - 1.000) |
| 1. Treatment #1. Time | 0.903 | (0.517 - 1.579) | 0.511** | (0.262 - 0.995) | 0.371*** | (0.176 - 0.785) |
| 0b. Treatment #0b. Time #co. Education | 1.000 | (1.000 - 1.000) | 1.000 | (1.000 - 1.000) | 1.000 | (1.000 - 1.000) |
| 0b. Treatment #1o. Time #co. Education | 1.000 | (1.000 - 1.000) | 1.000 | (1.000 - 1.000) | 1.000 | (1.000 - 1.000) |
| 1o. Treatment #0b. Time #co. Education | 1.000 | (1.000 - 1.000) | 1.000 | (1.000 - 1.000) | 1.000 | (1.000 - 1.000) |
| 1. Treatment #1. Time #c. Education | 1.962* | (0.991 - 3.887) | 1.545 | (0.534 - 4.470) | 1.047 | (0.351 - 3.125) |
| **Age group (compared with 15-19)** | | | | | | |
| 20-34 | 1.247 | (0.780 - 1.994) | 0.911 | (0.533 - 1.558) | 1.201 | (0.550 - 2.621) |
| 35-49 | 1.181 | (0.721 - 1.932) | 0.854 | (0.453 - 1.609) | 1.146 | (0.496 - 2.649) |
| **Marital Status (compared with Single)** | | | | | | |
| Married | 0.992 | (0.685 - 1.438) | 0.734* | (0.527 - 1.023) | 1.011 | (0.571 - 1.792) |
| **Wealth Index (compared with poor)** | | | | | | |
| Not poor | 1.133 | (0.916 - 1.401) | 1.191 | (0.909 - 1.560) | 0.933 | (0.694 - 1.254) |
| **Religion (compared with Christianity)** | | | | | | |
| Traditional African Religion | 0.908 | (0.639 - 1.291) | 0.919 | (0.656 - 1.286) | 0.816 | (0.555 - 1.198) |
| Islamic Religion | 0.710*** | (0.557 - 0.906) | 1.295* | (0.973 - 1.724) | 1.495** | (1.071 - 2.087) |
| **Location of Residence (compared with Urban)** | | | | | | |
| Semi-urban | 1.550 | (0.841 - 2.856) | 1.295 | (0.677 - 2.478) | 2.082* | (0.946 - 4.583) |
| Rural | 1.288 | (0.748 - 2.216) | 1.085 | (0.672 - 1.752) | 1.820* | (0.917 - 3.612) |
| **Parity (compared with one birth)** | | | | | | |
| 2 - 4 births | 1.105 | (0.836 - 1.460) | 0.785* | (0.589 - 1.044) | 1.122 | (0.751 - 1.674) |
| 5 - 7 births | 0.936 | (0.674 - 1.299) | 0.731 | (0.497 - 1.075) | 1.257 | (0.750 - 2.107) |
| 8 or more births | 0.915 | (0.506 - 1.657) | 0.777 | (0.405 - 1.490) | 1.050 | (0.432 - 2.554) |
|  | | | | | | |
| Constant | 0.258*** | (0.127 - 0.523) | 0.202*** | (0.091 - 0.450) | 0.034*** | (0.011 - 0.108) |
|  | | | | | | |
| Observations | 3,031 |  | 3,359 |  | 3,384 |  |

Robust cieform in parentheses

*** p<0.01, ** p<0.05, * p<0.1

**Table S4: Mean Comparison Test for Neonatal illness (t-test)**

|  | **Non-Intervention** | **Mean** | **Std Err** | **Pr(\|T\|>\|t\|)** | **Intervention** | **Mean** | **Std Err** | **Pr(\|T\|>\|t\|)** |
| --- | --- | --- | --- | --- | --- | --- | --- | --- |
| **Poor** | Baseline | 0.293 | 0.013 | <0.001 | Baseline | 0.312 | 0.013 | <0.001 |
|  | Endline | 0.164 | 0.009 |  | Endline | 0.167 | 0.009 |  |
| **Non poor** | Baseline | 0.293 | 0.013 | <0.001 | Baseline | 0.321 | 0.013 | <0.001 |
|  | Endline | 0.180 | 0.009 |  | Endline | 0.165 | 0.008 |  |
|  | | | | | | | | |
| **No Education** | Baseline | 0.285 | 0.012 | <0.001 | Baseline | 0.292 | 0.011 | <0.001 |
|  | Endline | 0.158 | 0.008 |  | Endline | 0.166 | 0.008 |  |
| **Some Education** | Baseline | 0.306 | 0.013 | <0.001 | Baseline | 0.330 | 0.014 | <0.001 |
|  | Endline | 0.178 | 0.009 |  | Endline | 0.169 | 0.009 |  |

**Table S5: Mean Comparison Test for Diarrhea (t-test)**

|  | **Non-Intervention** | **Mean** | **Std Err** | **Pr(\|T\|>\|t\|)** | **Intervention** | **Mean** | **Std Err** | **Pr(\|T\|>\|t\|)** |
| --- | --- | --- | --- | --- | --- | --- | --- | --- |
| **Poor** | Baseline | 0.146 | 0.009 | 0.049 | Baseline | 0.158 | 0.009 | <0.001 |
|  | Endline | 0.123 | 0.008 |  | Endline | 0.111 | 0.007 |  |
| **Non poor** | Baseline | 0.159 | 0.009 | 0.017 | Baseline | 0.172 | 0.010 | <0.001 |
|  | Endline | 0.130 | 0.008 |  | Endline | 0.110 | 0.007 |  |
|  | | | | | | | | |
| **No Education** | Baseline | 0.154 | 0.008 | 0.001 | Baseline | 0.155 | 0.008 | <0.001 |
|  | Endline | 0.120 | 0.007 |  | Endline | 0.113 | 0.007 |  |
| **Some Education** | Baseline | 0.148 | 0.009 | 0.050 | Baseline | 0.173 | 0.010 | <0.001 |
|  | Endline | 0.125 | 0.008 |  | Endline | 0.113 | 0.007 |  |

**Table S6: Mean Comparison Test for Fever (t-test)**

|  | **Non-Intervention** | **Mean** | **Std Err** | **Pr(\|T\|>\|t\|)** | **Intervention** | **Mean** | **Std Err** | **Pr(\|T\|>\|t\|)** |
| --- | --- | --- | --- | --- | --- | --- | --- | --- |
| **Poor** | Baseline | 0.092 | 0.007 | 0.174 | Baseline | 0.093 | 0.007 | 0.049 |
|  | Endline | 0.105 | 0.007 |  | Endline | 0.074 | 0.006 |  |
| **Non poor** | Baseline | 0.094 | 0.007 | 0.533 | Baseline | 0.100 | 0.007 | 0.014 |
|  | Endline | 0.100 | 0.007 |  | Endline | 0.076 | 0.006 |  |
|  | | | | | | | | |
| **NO Education** | Baseline | 0.092 | 0.006 | 0.480 | Baseline | 0.093 | 0.006 | 0.284 |
|  | Endline | 0.099 | 0.006 |  | Endline | 0.084 | 0.006 |  |
| **Some Education** | Baseline | 0.092 | 0.007 | 0.487 | Baseline | 0.098 | 0.008 | 0.009 |
|  | Endline | 0.099 | 0.007 |  | Endline | 0.072 | 0.006 |  |
